# Prevalence, Management, and Impact of Dysmenorrhea on the Lives of Nurse and Midwife Trainees in Northern Ghana

**DOI:** 10.1155/2023/8823525

**Published:** 2023-08-14

**Authors:** Abubakari Wuni, Brenda Abena Nyarko, Mudasir Mohammed Ibrahim, Issahaka Abdulai Baako, Iddrisu Sisala Mohammed, Camillus Buunaaisie

**Affiliations:** ^1^Department of Medicine for the Elderly (C6), Cambridge University Hospitals NHS Foundation Trust, Addenbrookes Hospital, Cambridge, UK; ^2^Nurses' and Midwives' Training College, Tamale, Ghana; ^3^Xiangya Nursing School, Central South University, Yuelu, China; ^4^37 Military Hospital, Neghelli Baracks, Liberation Road, Accra, Ghana

## Abstract

**Background:**

Dysmenorrhea is the most common gynecological problem affecting the majority of female students in the nursing profession today. They often experience severe pain that is not only incapacitating but also has a significant impact on their day-to-day college life, academic, and clinical performance.

**Aim:**

This study was conducted to assess the prevalence, management, and impact of dysmenorrhea on the lives of nurse and midwife trainees in northern Ghana.

**Methods:**

A descriptive cross-sectional design with a quantitative approach to data collection was employed to collect data from nurse and midwife trainees in three colleges of nursing and/or midwifery in the northern region of Ghana. A proportionate stratified random sampling technique was used to recruit 303 respondents for the study. After gaining permission from various institutions, data were collected by using a structured questionnaire from 13^th^ September to 28^th^ October, 2022. Stata (special edition) for Windows version 17.0 was used for the statistical analyses.

**Results:**

The study revealed a high prevalence of dysmenorrhea among female nursing students (66.7% and 95% CI: 0.611–0.720). More than half of the respondents (67.3%) experienced loss of appetite for food. The most common site of most intense pain was the pelvis and lower abdomen (98.0%). A greater proportion of students (65.8%) used antispastic drugs to reduce pain. The respondents' concentration in the classroom was greatly affected (77.2%) as well as normal physical activities (58.4%). A multivariable logistic regression analysis revealed that the odds of dysmenorrhea are 2.67 times higher when the duration of menstruation is 4-5 days (AOR = 1.82, 95% CI = 1.13–6.28, and *p* = 0.024) than a duration of 1–3 days. Having urinary tract infections was associated with 3.56 times higher odds of dysmenorrhea (AOR = 3.56, 95% CI = 0.98–12.86, and *p* = 0.053). Again, the odds of dysmenorrhea were also four times higher among respondents with a family history of the same condition (AOR = 4.05, 95% CI = 2.16–7.61, and *p* = 0.001).

**Conclusion:**

The current study revealed a high prevalence of dysmenorrhea among nurse and midwife trainees in the northern part of Ghana. The majority of the respondent experienced loss of appetite and intense pain in the pelvis and lower abdomen, and their concentration during lectures was also significantly affected. The most predominant nonpharmacological method used for reducing the pain was sleep and the application of warm objects on the abdomen.

## 1. Background of the Study

Dysmenorrhea is one of the most common gynecological conditions that affect young women and has a grave impact on their education, social activities, and quality of life [1, 2]. Dysmenorrhea accounts for an estimated 140 million missed work or school days each year. As a result, home remedies and self-medication with NSAIDs, naproxen sodium, and mefenamic acid are the most common methods used by women to alleviate menstrual discomfort [3–5]. The American College of Obstetricians and Gynecologists (ACOG) defines dysmenorrhea as the “pain associated with menstruation.” Dysmenorrhea was reported worldwide to affect between 15.8% and 89.5% of menstruating women, with 2% to 29% reporting severe pain [6].

More than half of women of all ages suffer from dysmenorrhea, and the prevalence rates of the condition vary by age and country. Japan (15.8%), India (79.7%), the United States (85.0%), Australia (88.0%), Nigeria (83.1%), and Ethiopia (85.1%) have reported its incidence [7, 8]. Menstruation is the cyclical shedding of the mucosal layer of the uterine endometrium that occurs during a woman's reproductive years and is accompanied by bleeding from the vagina [9]. Nearly, 90% of young women experience some degree of pain and distress during their menstruation, which is a common reason for frequent gynecological consultations [10, 11]. The most common symptom of dysmenorrhea is severe pain in the lower abdomen (sharp spasms), along with other symptoms such as sweating, fatigue, headache, backache, moodiness, irritability, tachycardia, constipation, nausea, vomiting, diarrhea, breast tenderness, and painful urination [12, 13].

There are two types of dysmenorrhea: primary and secondary dysmenorrhea [14]. Menstrual pain without pelvic disease is called primary dysmenorrhea. It typically does not occur until ovulatory menstrual cycles are established, which typically begin 6–12 months after menarche [15]. Secondary dysmenorrhea is menstrual pain caused by an underlying condition like endometriosis, pelvic inflammatory disease, or another similar condition. It can start years after first menarche [16, 17]. Primary dysmenorrhea has been linked in previous studies to reduce daily activity, social withdrawal, absenteeism from school or work, lower academic performance, and higher healthcare costs [18]. Younger age, low BMI, smoking, early menarche, nulliparity, prolonged or abnormal menstrual flow, premenstrual somatic complaints, previous suspected pelvic infections, psychological disturbance, and genetic influence are all factors that affect the prevalence and severity of dysmenorrhea [5, 8].

The profession of nursing is extremely emotionally taxing and stressful. Nursing professionals have to deal with a wide range of people, at different times of the day, in varied situations [19]. One way to describe nursing, the most important profession in the healthcare industry, is that it is “a process of action, reaction, interaction, and transaction in which nurses provide patients with medical care and then become affected by a variety of complex interactive factors, such as social, cultural, economic, and political circumstances.”

Dysmenorrhea is one of the most common gynecological conditions that make it harder for female nursing students to focus, take part, use their test-taking skills [20, 21], and to skip college class sessions [22]. It is evident that dysmenorrhea has a significant effect on their academic performance as well as their experiences [23].

Dysmenorrhea is very common among female students in Ghana. For instance, Ameade et al. [3] discovered 83.8 percent, and Acheampong et al. [24] found 68.1 percent among a large number of young women. However, the general public is still unaware of the social and economic effects of dysmenorrhea on female students [3].

Research on in-depth qualitative and quantitative studies of dysmenorrhea and coping techniques employed by nursing students with dysmenorrhea is lacking, particularly in Ghana [25]. It is common knowledge that nursing students' health and professional identity are negatively impacted by their exposure to unbearable situations. Nursing students' academic and clinical performance may be affected by dysmenorrhea. The current study was therefore designed to assess the prevalence, management, and impact of dysmenorrhea on the lives of nurse and midwife trainees in northern Ghana.

## 2. Materials and Methods

### 2.1. Study Design

A descriptive cross-sectional design with a quantitative approach to data collection was employed to obtain data from the trainees in three nursing and/or midwifery colleges in the northern region of Ghana.

### 2.2. Study Area and Population

The study was conducted in three colleges of nursing and/or midwifery. These were Nurses' and Midwives' Training College, Tamale and Yendi College of Health Sciences located in the northern region, and Damongo Nursing Training College located in the Savannah region. The northern and Savannah regions are among the sixteen regions, located in the northern part of Ghana and cover an area of 70,384 square kilometres or 31 percent of Ghana's landmarks.

These three training colleges are public nursing schools in northern Ghana accredited to train skillful, competent, and disciplined nurses and midwives for the provision of quality healthcare. Nurses' and Midwives' Training College, Tamale has a female student population of 872, the Yendi College of Health Sciences has a female student population of 142, and Damongo Nursing Training College has a female student population of 233.

#### 2.2.1. Inclusion and Exclusion Criteria

Female students who were within the reproductive age of 15–49 and have experienced mensuration, those who have been on the campus for more than one semester, and were willing and able to provide consent were included in the study. Female students who were ill and those on clinical grounds were excluded. In addition, trainees who had chronic medical conditions or used medication that may affect the menstrual cycle or pain perception such as thyroid disorders and neuropathic pain medication were excluded.

### 2.3. Sample Size and Sampling Procedure

A sample size of 303 respondents was used in the study. It was determined using the Yamane [26] sample size calculation formula for a finite population.

Also, to determine the number of respondents for each college, a proportionate sampling was employed which states that

(1) $$\begin{array}{c} \frac{sample\;size}{population\;size}\times stratum\;size\text{.} \end{array}$$

A proportionate stratified random sampling technique was used to recruit respondents for the study from each year group.

### 2.4. Data Collection Tool

A structured questionnaire was used for data collection in the study. The questionnaire was adopted from a previous study [27] and designed to fit the current study. The questionnaire had the following three sections:

  Section A: demographic information of respondents' twenty-one (21) items
  Section B: dysmenorrhea characteristics of the respondents' eighteen (18) items
  Section C: impact of dysmenorrhea on the quality of life ten (10) items. Each question was coded with a unique number to facilitate easy identification

### 2.5. Validity and Reliability of Tools

To guarantee the instrument content and face validity, the questionnaire received peer review from health experts in order to ensure that the questions reflected the study objectives. The research instrument was pretested with 20 respondents, and their feedback was used to modify questions which were ambiguous and also to correct grammar errors.

### 2.6. Data Collection Procedure

Data collection commenced after gaining permission from various institutions. The data collection period was from 13^th^ September to 28^th^ October, 2022, after permission was granted.

The respective institutions provided a list of nurse and midwife trainees who met the specified criteria. To ensure that each trainee had an equal chance of being chosen, the trainees were randomly assigned numbers using a random number generator. Selected trainees were gathered in a lecture hall and informed of the aims of the study. The researchers obtained verbal and written consent from the trainees to participate in the study.

The questionnaires were distributed to the trainees after their consent was obtained. The researchers made sure that the trainees understood the questions and had enough time to complete them without interfering with their school work, and the trainees were given up to 3 days to return the completed questionnaire. The completed questionnaires were sealed in envelopes after being reviewed for completeness. The confidentiality of the information provided by respondents was protected.

### 2.7. Statistical Analysis

Categorical data were summarized using frequencies and percentages, while continuous data were summarized using the mean and standard deviation. The prevalence of dysmenorrhea was summarized using a simple proportion and a 95% confidence interval. Bivariate and multivariable logistic regression models were used to determine the factors associated with the prevalence of dysmenorrhea. Confidence intervals were computed at a 95% confidence level, and a *p* value of 0.05 or less was considered statistically significant. Stata (special edition) for Windows version 17.0 was used for the statistical analyses.

### 2.8. Ethical Consideration

Approval was obtained from the institutions with reference numbers MOH/NMTC/51/334-22, YCHS/24/10/22, and NTC/A1/22/063 before conducting the study in the three colleges. The consenting process of respondents involved a verbal explanation of the purpose of the study. Emphasis was laid on the voluntary nature of participation and that they could withdraw at any time during the study. A written consent form was signed by the respondents who agreed to voluntarily participate to help facilitate the study. Privacy, anonymity, and confidentiality of the respondents were ensured throughout the data collection process.

## 3. Results

### 3.1. Characteristics of Respondents

In all, 303 students from three health training institutions participated in the study. The mean age of the students was 22.61 ± 2.35 years, with a majority of 80.2%, within the age group of 20–24 years. 91.4% were not married and only 7.6% had given birth. The majority, that is, 56.4%, had their first menstruation at the age of 12–14 years, with 77.6% having regular menstrual cycles. More than half, that is, 56.8%, have their period between 4 and 5 days, with 75.9% experiencing normal bleeding.

About 64.0% engaged in sexual activity, 89.5% had premenstrual syndrome, 54.5% engaged in regular physical activity, and 57.8% had a sleep duration of about 5–9 hours. More than one-third (37.0%) had a family history of dysmenorrhea (Table 1).

### 3.2. Prevalence of Dysmenorrhea among Nurses and Midwife Trainees

The majority, that is, 202 (66.7% and 95% CI: 0.611–0.720), of the respondents have experienced dysmenorrhea. Out of the 202 respondents who have experienced dysmenorrhea, the majority (51.5%) started experiencing it from the first menstruation, 64.3% have their pain begin on the first day of menstruation, 98% have pain in the pelvis and lower abdomen, 50.5% experienced severe pain, and 59.9% experienced the pain for two days or more (Table 2).

### 3.3. Factors Associated with the Prevalence of Dysmenorrhea among Nurse and Midwife Trainees

Table 3 shows both bivariate and multivariable logistic regression analyses showing the factors associated with the prevalence of dysmenorrhea among nurse and midwife trainees. At the bivariate level, marital status, childbearing, blood flow during menstruation, history of a urinary tract infection, premenstrual syndrome, and family history of dysmenorrhea were significantly associated with dysmenorrhea (*p* < 0.05).

The multivariable logistic regression analysis revealed that the odds of painful menstruation was 2.67 times higher when the duration of menstruation is 4–5 days (AOR = 1.82, 95% CI = 1.13–6.28, and *p* = 0.024) than when the duration of menstruation is 1–3 days. Having urinary tract infections was associated with 3.56 times higher odds of painful menstruation (AOR = 3.56, 95% CI = 0.98–12.86, and *p* = 0.05). Again, the odds of painful menstruation were also four times higher among respondents with a family history of painful menstruation (AOR = 4.05, 95% CI = 2.16–7.61, and *p* = 0.001).

### 3.4. Symptoms Associated with Dysmenorrhea

Table 4 shows the symptoms associated with dysmenorrhea. It revealed that the most prevalent symptom was the loss of appetite for food (67.3%), followed by fatigue/feeling dizzy (46.5%) and headache (42.6%). The most annoying symptom reported to accompany menstruation was fatigue (24.7%), followed by loss of appetite (24.3%).

### 3.5. Management of Dysmenorrhea among Nurse and Midwife Trainees

Table 5 shows that nearly half of the respondents (47.5%) do nothing to reduce the pain. However, 20.8% use pharmacological methods, 13.4% use nonpharmacological methods, and 18.3% use both pharmacological and nonpharmacological methods to reduce menstrual pains. The most predominant nonpharmacological method used for reducing menstrual pain is sleep (59.4%) followed by the application of liquids or hot objects on the abdomen (50.0%).

### 3.6. Impact of Dysmenorrhea on the Quality of Life

Table 6 shows that the majority of the respondents indicated that dysmenorrhea affects their quality of life (74.8%). More than half of them reported that they could not perform normal physical activities (58.4%), 53.5% felt tired, and 50.5% could not have a normal diet. Again, dysmenorrhea had a negative effect on the academic work of most students (62.4%) and their social life (54.0%). More than two-third of the students could not concentrate in class when having dysmenorrhea (77.2%).

## 4. Discussion

The aim of this cross-sectional study was to assess the prevalence, management, and impact of dysmenorrhea among nurse and midwife trainees. In this present study, the prevalence of dysmenorrhea among trainee nurses and midwives was 66.7%. This finding is similar to other research studies conducted in various countries such as 63.3% in southern Spain [16], 60% in Canada [28], and 70.6% in Saudi Arabia [29]. This finding is also similar to a study conducted in the southeastern part of Ghana 68.1% [24]. However, other studies conducted have reported a higher prevalence rate of dysmenorrhea such as 88% in Australia [30], 89.6% in Lebanon [31], 85.1% in Palestine [32], 84.2% in Lithuania [33], 84.1% in Italy [34], and 83.6% in northern Ghana [3].

There are a number of reasons that could account for this variation in prevalence rates across studies, such as the lack of a method to define dysmenorrhea that is universally recognized, the age group of females chosen for the study populations, and how respondents perceive pain [35] and stress [36].

The study revealed that the majority of the respondents (51.5%) started experiencing dysmenorrhea from the first menstruation, have their pain begin on the first day of menstruation, and have pain in the pelvis and lower abdomen. This finding is in line with the previous studies [13, 27, 37]. Our study also identified that more than 50% of the respondents experienced severe pain similar to the studies conducted in Bangladesh [38], but in the majority of the previous studies, respondents classified their pain as moderate [29, 34, 39] and some even mild [40]. The difference in pain intensity across studies could be attributed to differences in pain perception among respondents, as well as different assessments of pain intensity scales used to assess pain severity.

In this study, the odds of painful menstruation was 2.67 times higher when the duration of menstruation is 4-5 days than when the duration of menstruation is 1–3 days. A menstrual duration of 4-5 days was a factor associated with the prevalence of dysmenorrhea. Females who had a menstrual duration of 4-5 days were more likely to experience dysmenorrhea. This finding is consistent with a previous study showing that dysmenorrhea is higher in females with longer menstrual duration [37, 41]. The odds of dysmenorrhea was 3.56 times higher in respondents with urinary tract infections. This might happen when one starts menstruating and has UTIs at the same time, and one common symptom of both dysmenorrhea and UTIs is cramping pain in the lower abdomen, which may intensify menstrual pain. Dysmenorrhea appears to run in families, and the odds of dysmenorrhea are 4 times higher among respondents with a family history of painful menstruation. This is in line with previous research that found a link between a family history of predisposition and dysmenorrhea [35, 37, 42]. This could be explained by the fact that daughters of mothers with dysmenorrhea also experience dysmenorrhea, which is connected to a learned behavior from the mother. Again, it could be because of a similar lifestyle and way of life [37].

Dysmenorrhea may be accompanied by other symptoms affecting trainees' quality of life such as diarrhea, nausea, vomiting, headache, and dizziness [16]. In our study, the most common symptoms associated with dysmenorrhea were loss of appetite for food, fatigue/feeling dizzy, and headache. These symptoms have also been reported to be among the most common in respondents in other studies [21, 27, 32]. A study found that the most prevalent symptom (51.9%) associated with menstrual pain was loss of appetite for food [43].

As the majority of our respondents reported experiencing severe pain, it would have been expected that most would use various methods to reduce the pain. However, nearly half of the respondents (47.5%) do nothing to reduce the pain. This is consistent with other studies, where most of the respondents ignored their menstrual pain [3, 24]. In our study, 20.8% used pharmacological methods; out of this, the majority used antispasmodic drugs which is similar to a study conducted in Egypt where spasmolytic drugs were mostly used [43]. However, in our study, only a few trainees used the nonpharmacological method that had been reported in most previous studies [21, 44]. As a result, there is an urgent need for health education regarding nonpharmacological pain relief techniques for the management of dysmenorrhea for trainees. The most predominant nonpharmacological methods used for reducing menstrual pain were sleep (59.4%) and the application of liquids or hot objects on the abdomen (50.0%). Similar results about nonpharmacological methods have been reported in the literature [43].

Regarding the impact of dysmenorrhea, the majority (74.8%) of the respondents indicated that dysmenorrhea affects their quality of life. This finding is not surprising as most trainees revealed that they experience severe pain during menstruation. More than half of them reported they could not perform normal physical activities, felt tired, and could not have a normal diet. Also, dysmenorrhea had a negative effect on the academic work of most students and their social life. More than two-third of the students could not concentrate in class when having painful menstruation. All these could subsequently result in having a negative impact on the academic performance of trainees [10]. Our results confirm the conclusion of other reports [23, 27, 45].

### 4.1. Limitation

In the current study, responses from the respondents were based on past experiences of dysmenorrhea and introduced a risk of recall bias, so the findings of this study should be interpreted with caution. We were unable to distinguish between primary and secondary dysmenorrhea, and for this reason, period pain was treated as a whole. Also, the majority of the questions were closed-ended and limited the chances to explore deeper views or opinions from the respondents.

## 5. Conclusion

The finding from this current study revealed a high prevalence of dysmenorrhea among nurse and midwife trainees in the northern part of Ghana. The majority of the respondent experienced loss of appetite and intense pain in the pelvis and lower abdomen, and their concentration during lectures was also significantly affected. The most predominant nonpharmacological method used for reducing the pain was sleep and warm objects on the abdomen.

## Figures and Tables

**Table 1:** Characteristics of respondents.

Variables	Frequency (*n* = 303)	Percent (%)
*Age (years)*
Mean ± SD	22.61 ± 2.35	
<20	8	2.6
20–24	243	80.2
25+	52	17.16
*Marital status*
Single	277	91.4
Married	26	8.6
*Have given birth before*
No	280	92.4
Yes	23	7.6
*Age at first menstruation (years)*
9–11	13	4.3
12–14	171	56.4
15+	119	39.3
*Nature of menstrual cycles*
Regular	235	77.6
Irregular	68	22.4
*Duration of menstruation (days*)
1–3	39	12.9
4–5	172	56.8
>5	92	30.4
*Blood flow during menstruation*
Normal bleeding (7–10 pads used)	230	75.9
Abundant bleeding (>10 pads used)	45	14.9
Reduced bleeding (7 pads used)	28	9.2
*Respondents' history of some gynecological conditions* ^*∗*^
Gynecological surgeries	5	1.7
Genital malformation	2	0.7
Vaginal candidiasis	68	22.4
Urinary tract infections	27	8.9
*Respondents' previous diagnosis* ^*∗*^
Endometriosis	6	2.0
Polycystic ovary syndrome	2	0.7
Pelvic inflammatory disease	13	4.3
*Previously treated for Chlamydia trachomatis or Neisseria gonorrhoeae*
No	281	92.7
Yes	22	7.3
*Result of the Papanicolaou test*
Normal	34	11.2
Inflammation	3	1.0
Never took this test	266	87.8
*Sexual activity*
Active	194	64.0
Inactive	109	36.0
*Contraceptive use*
Oral contraceptives	86	28.4
IUD	4	1.3
Coitus interruptus	25	8.3
*Pain during sexual intercourse*
No	239	78.9
Yes	64	21.1
*Sexual intercourse during menstruation*	
No	276	91.1
Yes	7	2.3
Occasionally	20	6.6
*Premenstrual syndrome*
No	35	11.5
Yes	229	75.6
Occasionally	39	12.9
*Regular physical activity*
No	138	45.5
Yes	165	54.5
*Sleep duration (hours)*
<5	107	35.3
5–9	175	57.8
10+	21	6.9
*Family history of dysmenorrhea*
No	191	63.0
Yes	112	37.0

Source: field data (2022).

**Table 2:** Prevalence of dysmenorrhea among nurse and midwife trainees.

Variables	Frequency	Percent (%)
*Experiences painful menstruation (dysmenorrhea)*		
No	101	33.3
Yes	202	66.7
*Onset of dysmenorrhea* (*n* = 202)		
From the first period	104	51.5
1 year after first menstruation	40	19.8
1–3 years after	25	12.4
4 years or more	33	16.3
*When does pain start*		
First day of menstruation	130	64.3
Day before menstruation	43	21.3
More than two days before menstruation	29	14.4
*Frequency of pain occurrence*		
<35% of menstrual cycles	54	26.7
35–70% of menstrual cycles	61	30.2
>70% of menstrual cycles	18	8.9
At each period	69	34.2
*Location of most intense pain*		
Pelvis and lower abdomen	198	98.0
Lumbar level	1	0.5
Thighs	3	1.5
*Intensity of pain*		
Easy	4	2.0
Moderate	96	47.5
Severe	102	50.5
*Experience intense pains during a stressful period*		
No	51	25.2
Yes	151	74.8
*Intensity of pain has reduced since the beginning of sexual life*		
No	169	83.7
Yes	33	16.3
*Duration of pain*		
Few hours	29	14.4
One hour	52	25.7
Two days or more	121	59.9

^*∗*^Source: field data (2022).

**Table 3:** Factors associated with the prevalence of dysmenorrhea among nurse and midwife trainees.

Variables	COR	95% CI	*p* value	AOR^*∗*^	95% CI	*p* value
*Age (years)*						
<20	1					
20–24	0.72	0.14–3.64	0.690			
25+	0.45	0.08–2.47	0.361			
*Marital* status						
Single	1			1		
Married	0.33	0.14–0.75	0.008	0.59	0.17–2.03	0.404
*Have given birth before*						
No	1			1		
Yes	0.29	0.12–0.69	0.006	0.39	0.10–1.46	0.166
*Age at first menstruation (years)*						
9–11	1			1		
12–14	0.17	0.02–1.39	0.099	0.19	0.23–1.65	0.134
15+	0.14	0.02–1.09	0.060	0.17	0.02–1.46	0.107
*Nature of menstrual cycles*						
Regular	1					
Irregular	0.76	0.43–1.33	0.331			
*Duration of menstruation (days)*						
1–3	1			1		
4-5	1.82	0.90–3.69	0.096	2.67	1.13–6.28	0.024
>5	1.96	0.91–4.23	0.087	2.31	0.90–5.88	0.080
*Amount of blood flow during menstruation*						
Normal bleeding (7–10 pads used)	1			1		
Abundant bleeding (>10 pads used)	2.76	1.23–6.21	0.014	1.83	1.14–6.28	0.192
Reduced bleeding (7 pads used)	1.79	0.73–4.39	0.202	2.13	0.78–5.83	0.140
*Respondents' history of some gynecological conditions * ^*∗∗*^						
Gynecological surgeries	2.02	0.22–18.31	0.532			
Genital malformation	0.50	0.03–8.03	0.623			
Vaginal candidiasis	1.52	0.83–2.77	0.175			
Urinary tract infections	4.40	1.29–14.99	0.018	3.56	0.98–12.86	0.053
*Previously treated for Chlamydia trachomatis or Neisseria gonorrhoeae*						
No	1					
Yes	1.76	0.63–4.93	0.279			
Sexual activity						
Active	1					
Inactive	1.63	0.97–2.72	0.064	1.21	0.67–2.18	0.516
*Pain during sexual intercourse*						
No						
Yes	1.50	0.81–2.77	0.198			
*Premenstrual syndrome*						
No	1			1		
Yes	2.28	1.11–4.70	0.025	1.78	0.79–3.97	0.162
Occasionally	1.22	0.49–3.06	0.668	1.13	0.41–3.19	0.804
*Regular physical activity*						
No	1			1		
Yes	1.52	0.94–2.46	0.087	1.61	0.93–2.77	0.089
*Sleep duration (hours)*						
<5	1					
5–9	1.18	0.71–1.97	0.517			
10+	0.58	0.23–1.49	0.260			
*Family history of dysmenorrhea*						
No	1					
Yes	2.68	2.08–6.51	0.001	4.05	2.16–7.61	0.001

COR = crude odds ratio; AOR = adjusted odds ratio. ^*∗*^All variables were adjusted in the multivariable model. ^*∗∗*^Reference group. Source: field data (2022).

**Table 4:** Symptoms associated with dysmenorrhea.

Variables	Frequency (*n* = 202)	Percent (%)
*Symptoms associated with dysmenorrhea* ^*∗*^		
Vomiting	31	15.4
Headache	86	42.6
Sweating	37	18.3
Loss of appetite for food	136	67.3
Insomnia	36	17.8
Fatigue/feeling dizzy	94	46.5
Agitation/irritability	57	28.2
Only the pain appears	31	15.4
*Most annoying symptom accompanying pain*		
Vomiting	23	11.4
Headache	28	13.9
Sweating	8	4.0
Loss of appetite for food	49	24.3
Insomnia	14	6.9
Fatigue/feeling dizzy	50	24.7
Agitation/irritability	30	14.8

^*∗*^Multiple responses allowed. Source: field data (2022).

**Table 5:** Management of dysmenorrhea among nurse and midwife trainees.

Variables	Frequency (*n* = 202)	Percent (%)
*Methods used to reduce pain*		
Pharmacological methods	42	20.8
Nonpharmacological methods	27	13.4
Both	37	18.3
Nothing to reduce pain	96	47.5
*Type of medicine used to reduce pain* (*n* = 79)		
Nonsteroidal anti-inflammatory drugs		
Antispasmodics	52	65.8
Both	9	11.4
Does not use medicine	12	15.2
Other	6	7.6
*Time respondents start taking medication* (*n* = 79)^*∗*^		
Before pain appears	17	21.5
Appearance of pain regardless of its intensity	15	19.0
When pain becomes unbearable	55	69.6
*Nonpharmacological methods to reduce pain* (*n* = 64)^*∗*^		
Consumption of sweets	5	7.8
Walk	11	17.2
Sleep	38	59.4
Massage of painful regions	24	37.5
Application of liquids or hot objects on the abdomen	32	50.0

^*∗*^Multiple responses allowed. Source: field data (2022).

**Table 6:** Impact of dysmenorrhea on the quality of life.

Variables	Frequency (*n* = 303)	Percent (%)
*Dysmenorrhea affects quality of life*		
No	51	25.2
Yes	151	74.8
*Change clothing style when menstruating*		
No	44	21.8
Yes	158	78.2
*Feels increased affection during menstruation*		
No	110	54.5
Yes	92	45.5
*Negative effect of dysmenorrhea on quality of life* ^*∗*^		
Feel more agitated or more nervous	71	35.2
Feel more tired	108	53.5
Cannot have a normal diet	102	50.5
Cannot perform normal physical activities	118	58.4
*Negative effect of dysmenorrhea on the student's relationship* ^*∗*^		
Affects social life	109	54.0
Relationship with family members	48	23.8
Partner relationship	70	34.7
Relationship with friends	80	39.6
Academic work	126	62.4
*Effect of dysmenorrhea on college performance* ^*∗*^		
Cannot concentrate in class	156	77.2
Failed exams	6	3.0
Individual study is affected	110	54.5
*Dysmenorrhea affects school activities*		
No	26	12.9
Yes, most of the time	103	51.0
Yes, occasionally	73	36.1
*Number of days one misses classes*		
Does not miss class	130	64.4
One day	42	20.8
2-3 days	22	10.9
More than 3 days	8	4.0

^*∗*^Multiple responses allowed. Source: field data (2022).

## Data Availability

The data used to support the findings of the study are available from the corresponding author upon request.

## References

[1] Alghamdi F., Al-Zahrani A., Alabdulaziz H. (2019). Associated factors and outcomes of dysmenorrhea among female nursing students at king abdulaziz university. *American Journal of Nursing Science*.

[2] Ahmed Omar A., Hossain M. S., Ali Abdi H., Ali Mohamud I. (2021). Prevalence of dysmenorrhea among female students attending salaam university in mogadishu, Somalia and factors associated with it. *Education, Sustainability & Society*.

[3] Ameade E. P. K., Amalba A., Mohammed B. S. (2018). Prevalence of dysmenorrhea among University students in Northern Ghana; its impact and management strategies. *BMC Women’s Health*.

[4] Chaurasia L., Shah L., Paudel G., Sarraf D., Shah P., Singh J. K. (2021). Self-medication practice in primary dysmenorrhea among nursing students: a cross sectional study. *MedS Alliance Journal of Medicine and Medical Sciences*.

[5] Assefa N., Demissie A., Hailemeskel S. (2016). Primary dysmenorrhea magnitude, associated risk factors, and its effect on academic performance: evidence from female university students in Ethiopia. *International Journal of Women’s Health*.

[6] Takhelchangbam N., Agarwal T., Saxena D. (2021). Prevalence of dysmenorrhea, its effect on class attendance and treatment pattern among medical, nursing and para-medical female students of a university in etawah district. *International Journal of Healthcare Education & Medical Informatics*.

[7] Conney C. S., Kretchy I. A., Asiedu-Danso M., Allotey-Babington G. L. (2019). Complementary and alternative medicine use for primary dysmenorrhea among senior high school students in the western region of Ghana. *Obstetrics and Gynecology International*.

[8] Manjunath S. M., Lakshmipathi B. S., Ravi Kumar S., Srinivasa V. (2020). Prevalence and treatment patterns of dysmenorrhea among female medical students: a questionnaire based study at KIMS, Koppal. *International Journal of Basic & Clinical Pharmacology*.

[9] Pokhrel M., Thapa M. (2021). Dysmenorrhea among nursing staff in a tertiary care center: a descriptive cross-sectional study. *JNMA; journal of the Nepal Medical Association*.

[10] Fernández-Martínez E., Onieva-Zafra M. D., Abreu-Sánchez A., Fernández-Muñóz J. J., Parra-Fernández M. L. (2019). Absenteeism during menstruation among nursing students in Spain. *International Journal of Environmental Research and Public Health*.

[11] Molla A., Duko B., Girma B. (2022). Prevalence of dysmenorrhea and associated factors among students in Ethiopia: a systematic review and meta-analysis. *Women’s Health*.

[12] Durand H., Monahan K., McGuire B. E. (2021). Prevalence and impact of dysmenorrhea among university students in Ireland. *Pain Medicine*.

[13] Mesele T. T., Dheresa M., Oljira L., Wakwoya E. B., Gemeda G. M. (2022). Prevalence of dysmenorrhea and associated factors among haramaya university students, eastern Ethiopia. *International Journal of Women’s Health*.

[14] Dahlawi H., Bukhari I., Alshammari F. (2021). Effect of dysmenorrhea on the academic performance among students studying in princess nourah bint abdulrahman university, riyadh. *International Journal of Medicine in Developing Countries*.

[15] Guimarães I., Póvoa A. M. (2020). Primary dysmenorrhea: assessment and treatment. *Revista Brasileira de Ginecologia e Obstetrícia/RBGO Gynecology and Obstetrics*.

[16] Abreu-Sánchez A., Parra-Fernández M. L., Onieva-Zafra M. D., Ramos-Pichardo J. D., Fernández-Martínez E. (2020). Type of dysmenorrhea, menstrual characteristics and symptoms in nursing students in southern Spain. *Healthcare*.

[17] Karanth S., Liya S. R. (2018). Prevalence and risk factors for dysmenorrhoea among nursing student and its impact on their quality of life. *International Journal of Reproduction, Contraception, Obstetrics and Gynecology*.

[18] Azagew A. W., Kassie D. G., Walle T. A. (2020). Prevalence of primary dysmenorrhea, its intensity, impact and associated factors among female students’ at Gondar town preparatory school, Northwest Ethiopia. *BMC Women’s Health*.

[19] Jennings B. M., Hughes R. G. (2008). Work stress and burnout among nurses: role of the work environment and working conditions. *Patient Safety and Quality: An Evidence-Based Handbook for Nurses*.

[20] Al-Zahrani A., Alabdulaziz H., Alghamdi F. (2018). The impact of dysmenorrhea on academic performance among female nursing students at king. *Abdulaziz University*.

[21] Armour M., Parry K., Manohar N. (2019). The prevalence and academic impact of dysmenorrhea in 21,573 young women: a systematic review and meta-analysis. *Journal of Women’s Health*.

[22] Syed A., Rao S. B. (2020). Prevalence of premenstrual syndrome and dysmenorrhea among medical students and its impact on their college absenteeism. *International Journal of Reproduction, Contraception, Obstetrics and Gynecology*.

[23] Fernández-Martínez E., Onieva-Zafra M. D., Parra-Fernández M. L. (2019). The impact of dysmenorrhea on quality of life among Spanish female university students. *International Journal of Environmental Research and Public Health*.

[24] Acheampong K., Baffour-Awuah D., Ganu D. (2019). Prevalence and predictors of dysmenorrhea, its effect, and coping mechanisms among adolescents in shai osudoku district, Ghana. *Obstetrics and Gynecology International*.

[25] Aziato L., Dedey F., Clegg-Lamptey J. N. A. (2015). Dysmenorrhea management and coping among students in Ghana: a qualitative exploration. *Journal of Pediatric and Adolescent Gynecology*.

[26] Yamane T. (1967). *Statistics: An Introductory Analysis*.

[27] Sima R. M., Sulea M., Radosa J. C. (2022). The prevalence, management and impact of dysmenorrhea on medical students’ lives—a multicenter study. *Healthcare*.

[28] Burnett M. A., Antao V., Black A. (2005). Prevalence of primary dysmenorrhea in Canada. Journal of obstetrics and gynaecology Canada: jogc. *Journal of Obstetrics and Gynaecology Canada*.

[29] Alsaleem M. A. (2018). Dysmenorrhea, associated symptoms, and management among students at King Khalid University, Saudi Arabia: an exploratory study. *Journal of Family Medicine and Primary Care*.

[30] Subasinghe A. K., Happo L., Jayasinghe Y. L., Garland S. M., Gorelik A., Wark J. (2016). Prevalence and severity of dysmenorrhoea, and management options reported by young Australian women. *Australian Family Physician*.

[31] Kabbara R., Ziade F., Gannagé-Yared M. H. (2014). Prevalence and etiology of menstrual disorders in Lebanese university students. *International Journal of Gynecology & Obstetrics*.

[32] Abu Helwa H. A., Mitaeb A. A., Al-Hamshri S., Sweileh W. M. (2018). Prevalence of dysmenorrhea and predictors of its pain intensity among Palestinian female university students. *BMC Women’s Health*.

[33] Vilšinskaitė D. S., Vaidokaitė G., Mačys Ž., Bumbulienė Ž. (2019). The risk factors of dysmenorrhea in young women. *Wiadomosci Lekarskie (Warsaw, Poland: 1960)*.

[34] Grandi G., Ferrari S., Xholli A. (2012). Prevalence of menstrual pain in young women: what is dysmenorrhea?. *Journal of Pain Research*.

[35] Karout S., Soubra L., Rahme D., Karout L., Khojah H. M. J., Itani R. (2021). Prevalence, risk factors, and management practices of primary dysmenorrhea among young females. *BMC Women’s Health*.

[36] Pakpour A. H., Kazemi F., Alimoradi Z., Griffiths M. D. (2020). Depression, anxiety, stress, and dysmenorrhea: a protocol for a systematic review. *Systematic Reviews*.

[37] Joshi T., Kural M., Noor N., Pandit D., Patil A. (2015). Menstrual characteristics and prevalence of dysmenorrhea in college going girls. *Journal of Family Medicine and Primary Care*.

[38] Haque S. E., Rahman M., Itsuko K., Mutahara M., Sakisaka K. (2014). The effect of a school-based educational intervention on menstrual health: an intervention study among adolescent girls in Bangladesh. *BMJ Open*.

[39] Al-Kindi R., Al-Bulushi A. (2011). Prevalence and impact of dysmenorrhoea among Omani high school students. *Sultan Qaboos Univ Med J*.

[40] Singh A., Kiran D., Singh H., Nel B., Singh P., Tiwari P. (2008). Prevalence and severity of dysmenorrhea: a problem related to menstruation, among first and second year female medical students. *Indian Journal of Physiology & Pharmacology*.

[41] Unsal A., Ayranci U., Tozun M., Arslan G., Calik E. (2010). Prevalence of dysmenorrhea and its effect on quality of life among a group of female university students. *Upsala Journal of Medical Sciences*.

[42] Ozder A., Salduz Z. (2020). The prevalence of dysmenorrhea and its effects on female university students’ quality of life: what can we do in primary care?. *Upsala Journal of Medical Sciences*.

[43] Mohamed H., Mansour S. E. (2013). The effect of dysmenorrhea on quality of life of technical secondary schools girls. *The Medical Journal of Cairo University*.

[44] Chen L., Tang L., Guo S., Kaminga A. C., Xu H. (2019). Primary dysmenorrhea and self-care strategies among Chinese college girls: a cross-sectional study. *BMJ Open*.

[45] Al-Jefout M., Seham A. F., Jameel H. (2015). Dysmenorrhea: prevalence and impact on quality of life among young adult Jordanian females. *Journal of Pediatric and Adolescent Gynecology*.

